# Efficacy, safety and regulatory status of SGLT2 inhibitors: focus on canagliflozin

**DOI:** 10.1038/nutd.2014.40

**Published:** 2014-11-03

**Authors:** B Haas, N Eckstein, V Pfeifer, P Mayer, M D S Hass

**Affiliations:** 1 Federal Institute for Drugs and Medical Devices, Bonn, Germany; 2 Applied Pharmacy, University of Applied Sciences Kaiserslautern, Campus Pirmasens, Carl-Schurz-Str. 10–16, Pirmasens, Germany

## Abstract

Prevalence of diabetes mellitus is inc^6^reasing, with a burden of 382 million patients worldwide at present (more than the entire US population). The International Diabetes Federation anticipates an increase up to 592 million patients by 2035. Another major problem arises from the fact that just 50% of patients with type 2 diabetes mellitus are at target glycaemic control with currently available medications. Therefore, a clear need for new therapies that aim to optimize glycaemic control becomes evident. Renal sodium-linked glucose transporter 2 inhibitors are new antidiabetic drugs with an insulin-independent mechanism of action. They pose one remarkable advantage compared with already established antidiabetics: increasing urinary glucose excretion without inducing hypoglycaemia, thereby promoting body weight reduction due to loss of ~300 kcal per day. This review focuses on canagliflozin, which was the first successful compound of this class to be approved by both the US Food and Drug Administration and the European Medicines Agency in 2013. Clinical trials showed promising results: enhancing glycaemic control was paralleled by reducing body weight and systolic and diastolic blood pressure. Nevertheless, some safety concerns remain, such as genital mycotic infections, urinary tract infections and cardiovascular risks in vulnerable patients, which will be closely monitored in several post-authorization safety studies.

## Introduction

Typical features of type 2 diabetes mellitus (T2DM) are insulin resistance of various organs such as liver, muscle and adipose tissue, abnormal hepatic glucose production and reduced glucose-stimulated insulin secretion.^1^ This panel of characteristics is caused at least in part by insensitivity of the insulin receptor and impairment of insulin signalling. In the early stages of developing T2DM, pancreatic insulin production increases to overcome resistance. However, during progression of T2DM, insulin secretion decreases owing to the depletion of pancreatic β-cells, resulting in absolute insulin deficiency and increase in plasma glucose levels.^2^ Long-term elevated plasma glucose levels are responsible for the development of microvascular complications, such as retino-, nephro- and neuropathy, and macrovascular complications, such as atherosclerosis, which are the most common long-term complications of T2DM.^3, 4^ Correcting insulin resistance and substituting insulin currently is regarded as the gold standard of diabetes therapy. In addition, several medications are available (Table 1), which improve glucose utilization and uptake into insulin-sensitive tissues^5^ such as metformin^6^ and rosi- and pioglitazone.^7^ Release of insulin from pancreatic stores is achieved by sulphonylureas^8^ or incretin mimetics such as glucagon-like peptide 1 analogues and dipeptidyl peptidase 4 inhibitors.^9^ A major disadvantage of most of these interventions is that the daily dietary calorie intake usually stays too high and, thus, progression of T2DM is supported.^10^ In addition, only 50% of patients with T2DM reach glycaemic control with currently available therapy options.^11, 12^ Many of the current T2DM treatments have dose-limiting safety or tolerability issues, including hypoglycaemia (sulphonylureas), oedema (glitazones), weight gain (sulphonylureas, glitazones) or gastrointestinal adverse events (glucagon-like peptide 1 analogues). Therefore, a medical need for therapies with lesser side effects, which in addition increase glycaemic control, becomes evident. These considerations led to the clinical development of a new class of antidiabetic drugs: inhibitors of the renal sodium-linked glucose transporter 2 (SGLT2).^13^ This approach aims at therapeutically induced glucose excretion with urine. It combines two medical needs: glycaemic control and reduction of already ingested calories (as glucose is secreted unmetabolized). For physicians, this is a new approach. Throughout the following, we review the molecular mechanism of action, regulatory status, efficacy and safety of SGLT2 inhibitors with focus on canagliflozin.

## Regulation of glucose homeostasis by the kidneys

The kidneys regulate the homeostasis of numerous substances of the body. In addition to the regulation of protein, mineral and acid–base balance, the kidneys have a crucial role in the control of energy homeostasis.^14^ Especially, the role of the kidneys in glucose metabolism is important and includes, in addition to gluconeogenesis and glucose utilization, glucose filtration and reabsorption.^15^ Owing to its low molecular weight, ingested glucose is filtered into primary urine and is recovered by the kidneys. Daily dietary glucose intake (~180 g) is filtered and recovered afterwards. This mechanism was important for survival in times of food scarcity. Glucose recovery is mediated by a tubular transport system that can reabsorb glucose in combination with sodium (sodium-linked glucose transporter 1 and 2, SGLT1 and 2, respectively).^16^ This mechanism is not a unique feature of renal tubules, but also exists in other organs, such as the intestine. Whereas in the gastrointestinal tract especially *SLC5A1* (SGLT1) is expressed, renal tubules express *SLC5A1* and *SLC5A2* (SGLT2).^17^ An overview of all known SGLT transporters and their tissue distribution is depicted in Table 2.

**Figure 1:**
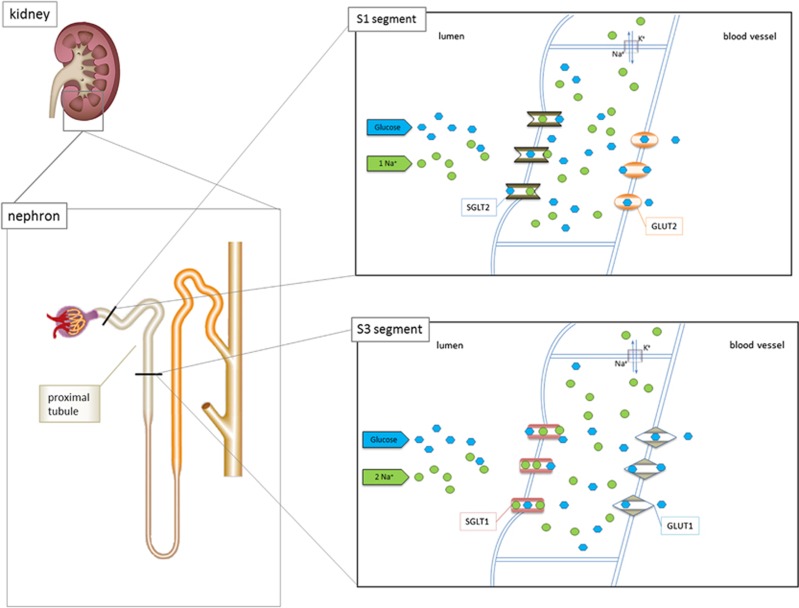
Schematic overview depicting the localization and function of SGLT1 and SGLT2 in the kidneys. SGLT2 reabsorbs glucose in combination with sodium in a 1:1 ratio in the tubular S1 segment, whereas SGLT1 reabsorbs glucose in combination with sodium in a 1:2 ratio in the tubular S3 segment. Both transporters are secondary active and driven by the activity of the Na^+^/K^+^-ATPase. Glucose reuptake into blood vessels is facilitated by glucose transporters GLUT1 and GLUT2.

Both transporters are able to reabsorb glucose. However, they show significant differences in affinities and transport capacity: SGLT2 has a greater transport capacity and reabsorbs glucose in combination with sodium in the ratio 1:1. SGLT1 has a higher affinity for glucose and reabsorbs glucose in combination with sodium in the ratio 1:2.^17, 18^ These different transport properties are used by the kidneys to reabsorb all energy, leading to glucose-free urine. SGLT2 is localized mainly in the first two segments of the proximal tubular system (S1 and S2 segment). Owing to its high transport capacity, it is capable of reabsorbing about 90% of glucose from the primary urine. Ten percent of initially filtered glucose is recovered in the third section of the proximal tubule (S3 segment) by SGLT1 because of its high affinity. Both transporters are secondarily active owing to their dependence on the activity of the Na^+^/K^+^-ATPase in the basolateral membrane for the active removal of sodium. Glucose transporters (GLUT2 and GLUT1) facilitate glucose transport across the basolateral membrane in the early and more distal regions of the proximal tubule^17^ (Figure 1).

There are certain mechanisms of compensation in cases of a surplus or failure of a transporter system. Diabetic patients, for example, have an increased glucose excretion in the primary urine. In this case, *SLC5A2* expression is often increased, leading to an increase in the renal threshold for glucose in the final urine.^19^ As a consequence, the kidneys also retain body glucose under diabetic conditions. On the other hand, an inhibition of SGLT2 reduces glucose reabsorption in the S1 and S2 segments and can be compensated only in part by SGLT1 in the S3 segment so that complete reabsorption of glucose is not warranted.^20^ Under physiological circumstances, this dual mechanism of reabsorption allows the careful handling of an important energy source, namely glucose.

On the basis of this knowledge, a new therapeutic principle for the treatment of T2DM was developed.^13^ Inhibition of the SGLT transport system results in increased urinary glucose excretion due to reduced glucose reabsorption. As a consequence plasma glucose levels are lowered, resulting in loss of calories and antihyperglycaemic effects beneficial for patients with T2DM.

## Regulatory status of SGLT2 inhibitors

Different SGLT2 inhibitors, also called gliflozins, are currently marketed or are undergoing clinical development and approval procedures (Table 3). In November 2012, the first-in-class SGLT2 inhibitor, dapagliflozin (Forxiga), was granted a marketing authorization by the European Medicines Agency (EMA). Dapagliflozin was initially rejected by the US Food and Drug Administration (FDA) owing to concerns noted by an Advisory Panel about potential increases in the risk of bladder and breast cancers associated with the drug.^21^ In January 2014, FDA granted marketing authorization for dapagliflozin (Farxiga) after new safety data on dapagliflozin from ongoing studies were provided. The FDA required to perform several post-marketing studies, in which >17 000 patients will be followed for 4–5 years to clarify whether dapagliflozin therapy is associated with increased risks for cardiovascular (CV) events, liver toxicity or cancer. Other post-marketing studies are required by the FDA to further assess bladder cancer risk and dapagliflozin's effect in paediatric patients. Furthermore, an intense pharmacovigilance programme to monitor reports of liver toxicity and pregnancy outcomes has to be implemented by the company. Canagliflozin (Invokana) was approved by the FDA in March 2013, and in November 2013 by the EMA. Marketing authorizations followed a positive opinion of Advisory Committees, but concerns about safety of canagliflozin remained, which are addressed in several post-authorization safety studies (please also refer to the section on safety of SGLT2 inhibitors for details). In Europe, canagliflozin is labelled with an inverted black triangle in the package leaflet and the summary of product characteristics (SmPC),^22^ indicating that the drug is under additional monitoring by regulatory authorities. On March 2014, the EMA recommended granting of a marketing authorization for a third SGLT2 inhibitor empaglifozin (Jardiance), and in May 2014 the European Commission followed the positive vote of the EMA. Initially, FDA rejected approval of empagliflozin in March 2014 owing to previously observed deficiencies at a facility where empagliflozin is manufactured, but finally approved it in August 2014. In Japan four SGLT2 inihibitors, namely dapagliflozin, ipragliflozin, tofogliflozin and luseogliflozin, were approved in 2014 (Table 3).

## Pharmacokinetics of canagliflozin

For gliflozins, comprehensive data from phase II and III clinical trials are available.^23^

Throughout the following sections, we focus exemplarily on canagliflozin as the first SGLT2 inhibitor licensed in the US and Europe.

Canagliflozin has an oral bioavailability of 65% with a maximum effect after 30–120 min. Its pharmacokinetic profile is independent of age, body weight, gender, ethnicity, administration with food and mild-to-moderate hepatic impairment. Importantly, the amount of glucose filtered in the glomerulus depends not only on plasma glucose levels but also on the glomerular filtration rate (GFR). Therefore, it can be expected that with decreasing renal function a decrease of activity is paralleled. Unlike with other antidiabetic drugs, such as metformin or glimepiride, with impaired renal function there is no risk to the patient, but the treatment becomes gradually ineffective. As a consequence, canagliflozin therapy should not be initiated in patients with end-stage renal disease, on dialysis, or with renal impairment at an eGFR<60 ml^–1^ min^–1^ per 1.73 m^2^. Clinical trials^24^ confirmed that significant glycaemic efficacy still was observed in subjects with eGFR values of *⩾* 30 to <50 ml^–1^ min^–1^ per 1.73 m^2^, even though the reductions in glycosylated haemoglobin A_1c_ (HbA_1c_) and fasting plasma glucose were smaller than seen in subjects with higher baseline eGFR values (see section below). In canagliflozin-treated patients whose eGFR falls below 60 ml^–1^ min^–1^ per 1.73 m^2^, canagliflozin dose should be adjusted to or maintained at the low dose of 100 mg once daily. Approximately 40% of administered canagliflozin is excreted unmetabolized in faeces; minor metabolism of canagliflozin occurs via uridine 5'-diphospho-glucuronosyltransferase, resulting in two pharmacologically inactive metabolites (canagliflozin O-glucuronides) mainly excreted via urine. With a half-life of 11–13 h a once-daily regimen is therapeutically sufficient (SmPC Invokana^22^).

## Pharmacology of canagliflozin—effects on glucose metabolism, body weight and the CV system

Canagliflozin is a competitive, reversible, highly selective SGLT2 inhibitor with a 250-fold selectivity towards SGLT2 over SGLT1. Inhibition of SGLT2 by canagliflozin leads to a reduction of glucose reabsorption from primary urine. The induced glucosuria of ~70 g per day results in a loss of glucose and optimized plasma glucose control.^20, 25^ Canagliflozin is indicated in patients with T2DM to improve glycaemic control as monotherapy when diet and exercise alone do not provide adequate glycaemic control and in patients for whom the use of metformin is considered inappropriate owing to intolerance or contraindications. Furthermore, it is indicated as add-on therapy with other antihyperglycaemic medicinal products including insulin, when these, together with diet and exercise, do not provide adequate glycaemic control (SmPC Invokana^22^). Table 4 summarizes the efficacy data of canagliflozin derived from phase III clinical trials. Canagliflozin is used at doses of 100 or 300 mg and the primary end point in the pivotal studies was lowering HbA_1c_. Serving as secondary end points, effects on fasting plasma glucose, body weight, blood pressure and lipid parameters were investigated.

As reflected in the European Public Assessment Report (EPAR) of canagliflozin,^26^ 7803 subjects were randomized. This included 4994 subjects treated with canagliflozin (100 or 300 mg), 1583 treated with placebo and 1226 treated with an active comparator (744 sitagliptin, 482 glimepiride). Taken together, depending on the premedication and baseline HbA_1c_ placebo-adjusted reduction, between −0.57% and −0.91% (100 mg dose) and −0.70% and −1.16% (300 mg dose) was observed, respectively. In poorly controlled diabetic patients even a reduction of up to – 2.42% was achieved. The favourable effect on HbA_1c_ values was consistent with an improvement of secondary end points such as fasting plasma glucose. The efficacy of canagliflozin was reduced in patients with moderate renal impairment. A meta-analysis of all subjects from placebo-controlled phase III studies with eGFR⩾30 to <60 ml^–1^ min^–1^ per 1.73 m^2^ (1085 subjects) showed a decrease in HbA_1c_ from baseline by −0.47% and −0.38% for canagliflozin 300 and 100 mg, respectively, compared with placebo.^26^ This is in line with the mode of action (see sections above) and is reflected in the SmPC^22^ as a warning for patients with end-stage renal disease, on dialysis, or with renal impairment and an eGFR <60 ml^–1^ min^–1^ per 1.73 m^2^.

The improvement in glycaemic control in T2DM patients achieved by SGLT2 inhibition also led to the consideration to use SGLT2 inhibitors in conjunction with insulin therapy in type 1 diabetes mellitus. So far, only limited data from studies with type 1 diabetic patients are available. Studies with empagliflozin in streptozotocin-induced diabetic rats^27^ and results from a Phase II trial indicate beneficial effects of SGLT2 inhibition on reducing HbA_1c_, body weight, total daily insulin dose and hypoglycaemic events.^28^ Further studies with empagliflozin and other SGLT2 inhibitors are currently underway to prove their efficacy and safety in type 1 diabetes mellitus patients.

Canagliflozin therapy not only was effective in improving glucose metabolism. The induced glucosuria of 70 g per day additionally leads to an energy deficit of 300 kcal per day, which translates into a body weight reduction of −1.84 and −2.43 kg (100 and 300 mg canagliflozin, respectively).^26^ Interestingly, weight loss stabilizes after a couple of weeks, even so glucosuria persists and calorie loss is maintained. Compensatory mechanisms such as increased food intake might explain these findings. That weight loss was indeed caused by loss of fat mass and not osmotic diuresis could be shown by dual-energy X-ray absorptiometry body composition analysis in clinical trials,^29, 30^ a diagnostic technique that can distinguish between fat, bone mineral and other fat-free mass that does not include bone. The weight-reducing characteristics of SGLT2 inhibitors in T2DM patients also might be effective in non-diabetic overweight subjects. This not only creates the option to widen the indication for the use of SGLT2 inhibitors as antiobesity drugs, but also poses the risk for drug abuse, for instance, as a weight-reducing agent or doping compound.

Under canagliflozin therapy, a clinically relevant lowering of systolic (−3.9 and −5.3 mmHg for the 100 and 300 mg dose, respectively) and diastolic (−2.1 and −2.5 mm Hg for the 100 and 300 mg dose, respectively) blood pressure was observed in placebo-controlled studies, which is a desirable additional effect of canagliflozin and not usually expected for a glucose-lowering drug. This was not accompanied by an increased compensatory heart rate. Blood pressure reduction correlates with a mild diuretic effect and haemoconcentration (reflected by increased haemoglobin and haematocrit) observed during canagliflozin treatment.^22, 31^ Whether the renin–angiotensin–aldosterone system is counter-regulatory activated upon canagliflozin treatment as observed in patients with genetic loss of *SLC5A2* (familial renal glucosuria)^32^ is currently under investigation.

T2DM usually entails marked long-term clinical consequences such as blindness and neuropathies derived from micro- or macroangiopathy. The question whether these hazardous clinical consequences can be reduced by canagliflozin therapy is currently under investigation and can, thus, not be answered conclusively at present.

## Safety of canagliflozin

Among the most important safety aspects of antidiabetic drugs is their low propensity to induce hypoglycaemia. Interestingly, during clinical development of SGLT2 inhibitors hardly any hypoglycaemia was observed due to the following reasons: first, glucose excretion decreases along with decreasing plasma glucose levels and; second, counter-regulatory mechanisms are not affected by SGLT2 inhibition as gliflozins act independently from insulin. This low risk for hypoglycaemia can be regarded an advantage of this class of medicines as compared with classical antidiabetic compounds such as insulin or sulphonylureas.

The adverse events observed with canagliflozin reflect its pharmacodynamic action. Almost no organ toxicities were found during pre-clinical development;^26^ only in long-term rat studies toxicities in terms of excessive bone growth (hyperostosis) and renal tubular tumours were observed, probably caused by undesired SGLT1 inhibition in the gastrointestinal tract and subsequent carbohydrate malabsorption. However, this is regarded as a species-specific phenomenon, as in rats canagliflozin is poorly absorbed from the gut and hence high local concentrations are reached, which are able to inhibit SGLT1 located at the surface of the intestinal mucosa. Carbohydrate malabsorption is accompanied by increased calcium absorption from the gut, probably causing hyperostosis and renal tubular tumours in the rat.

Nevertheless, SGLT2 inhibition can lead to unexpected sequels. It appears logical that glucosuria, deliberately induced by SGLT2 inhibition, favours urinary tract infections, as glucose serves as nutrient for bacteria. Some increase in urinary tract infections was indeed observed in regulatory safety trials; however, serious cases were rare.^33^ Much more pronounced were canagliflozin-related increases in female mycotic genital infections. Infections are not serious in nature and easy to treat, but patients and prescribers should be aware of it. Another reported side effect of canagliflozin treatment is osmotic diuresis and subsequent water loss. However, unlike that observed during classical osmotic diuresis, where sodium is retained, SGLT2 inhibition causes sodium loss. This can be attributed to the fact that sodium is co-transported with glucose by SGLT2 (Figure 1). In fact, canagliflozin leads to a decrease in blood pressure and haemoconcentration, reflected by increased haemoglobin and haematocrit (increased levels of plasma lipoproteins could also be a consequence of it, see below), but notably no hypernatraemia as a trigger for thirst. The latter may explain why, in particular, elderly patients do not develop sufficient thirst to compensate for water loss and consequently tend to have dehydration, unstable blood pressure or syncope.^34, 35^ A rather sharp decrease in blood pressure can particularly occur at the beginning of therapy; later on, counter-regulatory mechanisms like reduction in glomerular perfusion minimize diuresis and blood pressure reduction. In order to avoid haemodynamic problems in vulnerable patients, canagliflozin therapy should be initiated with the lower dose of 100 mg per day (instead of 300 mg per day). Consequently, concomitant use of canagliflozin and loop diuretics is not recommended.^22^

Regarding kidney function, there was intensive discussion during the licensing procedures whether canagliflozin might damage kidneys, especially when kidney function is already impaired by diabetic nephropathy.^26^ The above-mentioned haemoconcentration observed under canagliflozin therapy also leads to an increase in serum creatinine, which is usually a marker for renal damage. However, it could be shown that its increase with canagliflozin treatment is fully reversible after cessation, so that renal damage can be excluded. Contrarily, canagliflozin may help protect kidneys because it appears to reduce GFR by reducing intra-glomerular pressure and hence might retard glomerular sclerosis. This possibility is currently under investigation.^36^

There was also a discussion on the cancer risk of SGLT2 inhibitors. In phase III studies with the first SGLT2 inhibitor that was applied for marketing authorization, dapagliflozin, a marked numerical imbalance in the cases of bladder cancer was observed between the dapagliflozin and comparator groups; however, the number of cases was low. From 5501 patients under dapagliflozin 9 (0.16%) suffered from bladder cancer, whereas in the placebo group 1 of 3184 patients was affected (0.03%).^21, 37^ Reassuringly, no increase in bladder cancer was found subsequently with the other SGLT2 inhibitors, so that a chance finding is most likely.^38^ Carcinogenicity studies in animals were conducted with all SGLT2 inhibitors under development. For dapagliflozin, no tumours were observed in these studies.^37^ Canagliflozin caused certain types of tumours (for example, renal tubular tumours) in rats, but mechanistic studies revealed that off-target inhibition of SGLT1 in these animals was the underlying mechanism.^26^

For canagliflozin, a long-term CV safety study is ongoing (CANVAS), which was originally planned to demonstrate CV safety in patients with increased risk for CV events (Table 4).^39^ According to the present knowledge from a meta-analysis of clinical trials including an interim analysis of the CANVAS study provided in the EPAR of canagliflozin,^26^ canagliflozin does not increase the overall CV risk. Notably, in patients without CV disease, canagliflozin-treated patients performed numerically better than the comparator therapies in respect to major CV events; the point estimate for the hazard ratio was 0.52, albeit with a broad 95% confidence interval, including 1.^26^ This positive effect (if confirmed) could be related to the blood pressure-lowering effect of canagliflozin. On the other hand, in patients with existing CV disease the decrease in blood pressure, which is most pronounced immediately after onset of therapy, could be hazardous and could hence abolish the positive effect. For instance, the incidence of non-fatal stroke was numerically increased in the canagliflozin groups; further data from the CANVAS trial will show whether this effect is true. Also relevant for assessing CV safety is the plasma lipid profile; it turned out that canagliflozin (and also other SGLT2 inhibitors) slightly increased the levels of both high-density lipoprotein and low-density lipoprotein cholesterol.^33^ This simultaneous increase of both lipoproteins might be a consequence of the observed haemoconcentration (see above) and probably does not imply increased CV risk.

Further information on CV risk and also on other safety issues, for example, long-term renal safety, safety in special populations, and—as far as possible—tumour incidence, is expected from ongoing trials.

## Conclusion

SGLT2 inhibitors are a promising new class of antidiabetic drugs currently approved in Europe, the US and Japan. They allow weight-reducing and effective glycaemic control combined with a low risk of hypoglycaemia. The medication can be used in any stage of the disease independently of existing co-medications. Furthermore, the use of SGLT2 inhibitors in addition to insulin therapy in type 1 diabetic patients is conceivable, for example, to improve their glycaemic profile and avoid hyperglycaemia. However, whether SGLT2 inhibitor therapy also improves the clinical consequences of diabetes, such as micro- or macrovascular late complications, cannot be answered at present owing to immature clinical data, and provides some uncertainty of this class of drugs. Caution with these drugs should be exercised in elderly patients (tendency to dehydration and urinary-, genital infections), patients with recurrent urinary tract or genital infections and patients with reduced renal function (reduced efficacy).

## Figures and Tables

**Table 1:** Overview of currently available antidiabetic medications

*Class of antidiabetic drug*	*Mode of action*	*Target organ/tissue*	*Marketed drugs*^a^
α-Glucosidase inhibitors	Competitive inhibition of α-glucosidase: reduction of enzymatic degradation of polysaccharids in the intestine and in consequence reduced glucose uptake	Intestine	Acarbose, Miglitol
Biguanides	AMPK activation and mGPD inhibition: inhibition of gluconeogenesis, increased glucose uptake and fatty-acid oxidation; additional effects: reduction of glucose absorption from intestine, increase of insulin sensitivity, appetite suppressant	e.g., liver, muscle, kidney	Metformin
Gliflozines (SGLT2 inhibitors)	SGLT2 inhibition: reduction of glucose reabsorption in kidney leads to increased glucose excretion via urine	Renal proximal tubule	Canagliflozin, Dapagliflozin, Empagliflozin
Glinides	Sulphonylurea analogues with different pharmacokinetics: glucose-independent insulin release from pancreatic β-cells	Pancreatic β-cells	Nateglinide, Repaglinide
Gliptines (DPP4 inhibitors)	Inhibition of DPP4: delay of enzymatic incretine degradation	e.g., kidney, intestine, lung, vascular walls, plasma	Alogliptin, Linagliptin, Saxagliptin, Sitagliptin, Vildagliptin
Incretine mimetics (GLP1 analogues)	GLP1 receptor agonists: increased glucose sensitivity of β-cells, increased insulin sensitivity in target tissues due to removal of glucose toxicity, decreased glucagon secretion from α-cells, delayed gastric emptying and increased feeling of satiety	e.g., insulin target tissues, pancreas, CNS	Albiglutide, Exenatide, Liraglutide, Lixisenatide
Sulphonylurea	Glucose-independent insulin release from pancreatic β-cells	Pancreatic β-cells	1st generation: Chlorpropamide, Tolbutamide 2nd generation: Glibenclamide, Gliclazide, Glipizide, Gliquidone 3rd generation: Glimepiride
Thiazolidinediones (Glitazones)	PPARγ agonists: insulin-sensitizing effect on liver, muscle and adipose tissue; increased adipocyte differentiation	Liver, muscle, adipose tissue, CNS	Pioglitazone, Rosiglitazone

Abbreviations: AMPK, 5' adenosine monophosphate-activated protein kinase; CNS, central nervous system; DPP4, dipeptidyl peptidase 4; GLP1, glucagon-like peptide 1; mGPD, glycerophosphate dehydrogenase; PPARγ, peroxisome proliferator-activated receptor γ SGLT2, sodium-linked glucose transporter 2.

a Most prominent drugs marketed in the United States and/or Europe.

**Table 2:** Substrates, substrate affinities and tissue distribution of *SCL5* genes and the respective SGLT/SMIT transporter

*Gene (transporter)*	*Substrate*	*Apparent affinity, K*_*0.5*_ *(mM)*	*Tissue expression*
*SLC5A1* (hSGLT1)	D-Glucose	0.5	Intestine, trachea, kidney, heart, brain, testis and prostate
	D-Galactose	1	
*SLC5A2* (hSGLT2)	D-Glucose	5	Kidney, brain, liver, thyroid, muscle and heart
	D-Galactose	>100	
*SLC5A3* (cSMIT1)	Myo-inositol	0.050	Brain, heart, kidney and lung
	D-Glucose	>50	
*SLC5A4* (hSGLT3)	D-Glucose, Miglitol	19, 0.003	Brain, intestine, kidney, lung, muscle, testis and uterus
*SLC5A9* (hSGLT4)	D-Glucose, D-Mannose	7.7, 0.15	Intestine, kidney liver, brain, lung, trachea, uterus and pancreas
*SLC5A10* (hSGLT5)	D-Glucose, D-Galactose	Not known	Kidney
*SLC5A11* (rtSMIT2, rtSGLT6)	Myo-inositol	0.27	Brain, kidney and intestine
	D-Glucose	36	

Abbreviations: c, canine; h, human; rt, rat.

Substrate specificity, apparent affinity (K_0.5_ for the substrates) and tissue distribution according to (16;17;40–47).

**Table 3:** Overview of the current regulatory status of SGLT inhibitors for the treatment of diabetes

*SGLT inhibitor*	*Trial registration number*	*Daily dose*	*Approval/developmental status*	*SGLT selectivity*
Dapagliflozin	NA	5, 10 mg	Approved by EMA (2012/11), FDA (2014/01), PMDA (2014/03)	SGLT2
Canagliflozin	NA	100, 300 mg	Approved by FDA (2013/03), EMA (2013/11)	SGLT2
Empagliflozin	NA	10, 25 mg	Approved by EMA (2014/05), FDA (2014/08)	SGLT2
Ipragliflozin	NA	25, 50 mg	Approved by PMDA (2014/01)	SGLT2
Tofogliflozin	NA	20 mg	Approved by PMDA (2014/3)	SGLT2
Luseogliflozin	NA	2.5, 5 mg	Approved by PMDA (2014/03)	SGLT2
Ertugliflozin	NCT01958671	5, 10 mg	Phase III recruiting	SGLT2
LX4211	NCT01742208	Not yet determined	Phase II	SGLT1/SGLT2
GSK189075	NCT00500331	Not yet determined	Phase II	SGLT2
EGT0001442	NCT01377844	Not yet determined	Phase II	SGLT2
BI 44847	NCT00558909	Not yet determined	Phase I	SGLT2
EGT0001474	NCT00924053	Not yet determined	Phase I	SGLT2
GSK-1614235	NCT01607385	Not yet determined	Phase I	SGLT1
ISIS-SGLT2Rx	NCT00836225	Not yet determined	Phase I	SGLT2

Abbreviations: EMA, European Medicines Agency; FDA, Food and Drug Administration; NA, not applicable; PMDA, Pharmaceuticals and Medical Devices Agency.

Japan; source of information: homepages of the FDA,^48^ EMA,^22^ PMDA,^49^ (www.clinicaltrials.gov).

**Table 4:** Efficacy data of canagliflozin in phase III clinical trials

*Trial registration number/study type*	*Design, duration (duration to primary end point/duration of extension phase)*	*Intervention*	*No. subjects per treatment arm (ITT)*	*Primary end point*	*Baseline HbA*_*1c*_ *(%), mean*±*s.d.*	*Change in HbA*_*1c*_ *from baseline (%), LS mean (SE)*
*Monotherapy*^a^						
NCT01081834,^50,51^ main study^b^ monotherapy (90 centres)	PC, PG 52 wks (26 wks/26 wks)	Placebo	192	Δ BL to wk 26 in HbA_1c_	7.97±0.955	0.14±0.065
		Canagliflozin 100mg	195		8.06±0.959	−0.77±0.065
		Canagliflozin 300mg	197		8.01±0.988	−1.03±0.064
	52 wks extension period	Canagliflozin 100mg	191	Δ BL to wk 52 in HbA_1c_	8.06±0.959	−0.75±0.067
		Canagliflozin 300mg	194		8.01±0.988	−1.04±0.067
High glycaemic substudy monotherapy (40 centres)	PG 26 wks (26 wks/no extension)	Canagliflozin 100mg	47	Δ BL to wk 26 in HbA_1c_	10.59±0.873	−2.13±0.220
		Canagliflozin 300mg	44		10.62±0.955	−2.56±0.227
						
*Add-on to AHA monotherapy*^a^						
NCT01106677b,^52^ add-on to metformin monotherapy (169 centres)	PC, AC, PG 52 wks (26 wks/26 wks)	Placebo	183	Δ BL to wk 26 in HbA_1c_	7.96±0.896	−0.17±0.060
		Canagliflozin 100mg	368		7.94±0.879	−0.79±0.044
		Canagliflozin 300mg	367		7.95±0.931	−0.94±0.044
		Sitagliptin 100mg	366		7.92±0.875	−0.82±0.044
	52-wks extension period	Canagliflozin 100mg	365	Δ BL to wk 52 in HbA_1c_	7.94±0.879	−0.73±0.047
		Canagliflozin 300mg	360		7.95±0.931	−0.88±0.047
		Sitagliptin 100mg	354		7.92±0.875	−0.73±0.047
NCT00968812,^30^ add-on to metformin monotherapy (157 centres)	AC, PG 104 wks (52 wks/52 wks)	Canagliflozin 100mg	483	Δ BL to wk 52 in HbA_1c_	7.78±0.787	−0.82±0.039
		Canagliflozin 300mg	485		7.79±0.779	−0.93±0.039
		Glimepiride (titrated from 1–6 or 8mg)	482		7.83±0.795	−0.81±0.039
						
*Add-on to dual-combination AHA therapy*^a^						
NCT01106625, add-on to metformin+sulphonylurea (85 centres)	PC, PG 52 (26 wks/26 wks)	Placebo	156	Δ BL to wk 26 in HbA_1c_	8.12±0.896	−0.13±0.075
		Canagliflozin 100mg	157		8.13±0.926	−0.85±0.075
		Canagliflozin 300mg	156		8.13±0.942	−1.06±0.076
	52-wks extension period	Placebo	150	Δ BL to wk 52 in HbA_1c_	8.12±0.896	−0.01±0.077
		Canagliflozin 100mg	155		8.13±0.926	−0.74±0.077
		Canagliflozin 300mg	152		8.13±0.942	−0.97±0.078
NCT01106690,^b^ add-on to metformin+pioglitazone (74 centres)	PC, PG 52 wks (26 wks/26 wks)	Placebo	115	Δ BL to wk 26 in HbA_1c_	8.00±1.010	−0.26±0.069
		Canagliflozin 100mg	113		7.99±0.940	−0.89±0.069
		Canagliflozin 300mg	114		7.84±0.911	−1.03±0.070
NCT01137812,^53^ add-on to metformin+sulphonylurea (140 centres)	AC, PG 52 wks (52 wks/no extension)	Canagliflozin 300mg	377	Δ BL to wk 52 in HbA_1c_	8.12±0.910	−1.03±0.048
		Sitagliptin 100mg	378		8.13±0.916	−0.66±0.049
						
*Special population studies*^a^						
NCT01106651,^29^ older adults (⩾55 to ⩽80 years of age) (90 centres)	PC, PG104 wks (26 wks/78 wks)	Placebo	237	Δ BL to wk 26 in HbA_1c_	7.76±0.785	−0.03±0.063
		Canagliflozin 100mg	241		7.77±0.773	−0.60±0.063
		Canagliflozin 300mg	236		7.69±0.779	−0.73±0.064
NCT01064414,^24^ moderate renal impairment (eGFR ⩾30 to <50 ml^–^^1^ min^–1^ per 1.73 m^2^) (89 centres)	PC, PG 52 wks (26 wks/26 wks)	Placebo	90	Δ BL to wk 26 in HbA_1c_	8.02±0.917	−0.03±0.090
		Canagliflozin 100mg	90		7.89±0.898	−0.33±0.090
		Canagliflozin 300mg	89		7.97±0.805	−0.44±0.089
	52-wks extension	Placebo	87	Δ BL to wk 52 in HbA_1c_	8.02±0.917	−0.07±0.104
	Period	Canagliflozin 100mg	89		7.88±0.886	−0.19±0.104
		Canagliflozin 300mg	89		7.97±0.805	−0.33±0.103
						
*Cardiovascular assessment study with efficacy substudies*^a^						
NCT01032629, cardiovascular study (369 centres)	PC, PG duration is event driven based on number of MACE events	Placebo	1441^c^	Assessment of hazard ratio for MACE events		
		Canagliflozin 100mg	1445^c^			
		Canagliflozin 300mg	1441^c^			
Insulin substudy (316 centres)	PC, PG 18 wks (18 wks/no extension)	Placebo	565	Δ BL to wk 18 in HbA_1c_	8.20±0.837	0.01±0.032
		Canagliflozin 100 mg	566		8.33±0.905	−0.63±0.031
		Canagliflozin 300 mg	587		8.27±0.894	−0.72±0.030
Sulphonylurea substudy (80 centres)	PC, PG 18 wks (18 wks/no extension)	Placebo	45	Δ BL to wk 18 in HbA_1c_	8.49±1.130	0.04±0.146
		Canagliflozin 100mg	42		8.29±0.831	−0.70±0.145
		Canagliflozin 300 mg	40		8.28±1.005	−0.79±0.147

Abbreviations: Δ, change from; AC, active-controlled; AHA, anti-hyperglycaemic agent; BL, baseline; eGFR, estimated glomerular filtration rate; ITT, intent-to-treat population; LS, least-squares; MACE, major adverse cardiovascular events; No., number; PC, placebo-controlled; PG, parallel group; QD, once daily; SU, sulphonylurea; wk(s), week(s).

Source of information: European Public Assessment Report (EPAR) of canagliflozin^26^ if not otherwise indicated.

a Double-blind and randomized.

b Subjects assigned to placebo were switched to sitagliptin during the double-blind extension period.

c Randomized and treated subjects (that is, safety analysis set).
